# Effect of Fe Content on the Microstructure Evolution and Deformation Mechanism of Warm-Rolled Cu-Fe Alloy

**DOI:** 10.3390/nano16140839

**Published:** 2026-07-08

**Authors:** Baosen Lin, Su Huang, Shuai Tang, Dongxiao Wang, Jianping Li

**Affiliations:** 1 State Key Laboratory of Digital Steel, Northeastern University, Shenyang 110819, China; 1810251@stu.neu.edu.cn (B.L.); 2010154@stu.neu.edu.cn (S.H.); tangshuai@ral.neu.edu.cn (S.T.); 2 School of Mechanical Engineering, Xijing University, Xi’an 710123, China; 20240209@xijing.edu.cn

**Keywords:** Cu-Fe alloy, warm rolling, Fe content, microstructure, deformation mechanism

## Abstract

Cu–Fe alloys combine the high electrical conductivity of Cu with the strengthening and magnetic contributions of Fe, making them promising high-strength, electrically conductive functional materials. However, for high-Fe Cu–Fe alloys with Fe contents exceeding 10 wt.%, the microstructural response, texture evolution, and two-phase deformation partitioning during warm rolling remain insufficiently understood. In this study, Cu–10Fe, Cu–15Fe, and Cu–20Fe alloys were investigated to clarify the effect of Fe content on microstructure evolution, texture characteristics, deformation behavior, and property balance after single-pass warm rolling at 500 °C with a 50% reduction. The results show that, as the Fe content increased from 10% to 20%, the Fe-rich phase became progressively denser after warm rolling and gradually transformed from discrete spherical/spindle-like particles into fibrous structures distributed along the rolling direction, while the average grain size of the alloy decreased. EBSD analysis indicates that increasing Fe content weakened the preferred orientation of the Cu matrix. The maximum texture intensity of the Cu matrix decreased from 5.08 to 4.21, and texture showed a weakening trend. The mechanical properties show that, with increasing Fe content, the ultimate tensile strength increased from 434 MPa to 514 MPa, whereas the elongation decreased from 10.7% to 5.1%. This indicates that the increased amount of Fe-rich phase enhanced strength but reduced plasticity; nevertheless, dynamic recovery and local recrystallization induced by warm rolling helped maintain a certain degree of ductility. The electrical conductivity decreased from 19.43% IACS to 16.71% IACS with increasing Fe content, corresponding to a decrease of only approximately 2.7% IACS, suggesting that warm rolling partially mitigated the negative effect of increasing Fe content on electrical conductivity. Based on the combined microstructural, texture, and KAM/GND analyses, the deformation behavior of the alloys with increasing Fe content exhibited a transition from heterogeneous deformation dominated by the Cu matrix/interface to cooperative deformation involving the Fe-rich phase.

## 1. Introduction

As metastable immiscible alloys, Cu–Fe alloys exhibit unique application potential in electronic packaging, 5G communication shielding materials, and rail-transit braking components owing to the high electrical and thermal conductivity of copper, together with the high strength, high hardness, and excellent magnetic properties of Fe [1,2,3,4,5]. According to the Fe content, Cu–Fe alloys are generally classified into low-Fe alloys (≤4 wt.%) and high-Fe alloys (≥4 wt.%) [6]. Among them, low-Fe alloys such as C19200 and C19400 have already been applied on an industrial scale. In recent years, with the continuous increase in shielding-performance requirements under high-frequency electromagnetic environments, high-Fe Cu–Fe alloys (>10 wt.%) have gradually become a research focus for next-generation high-performance electromagnetic shielding materials because of their significantly enhanced magnetic permeability and saturation magnetization [7,8,9,10].

At present, the mainstream preparation route for Cu–Fe alloy sheets and strips is cold rolling combined with multistage aging treatment [11,12,13,14]. Previous studies have shown that the ultimate tensile strength of a Cu–10Fe alloy can reach 543 MPa after 98% cold rolling, but its electrical conductivity decreases sharply to 13.5% IACS. This is mainly attributed to the high dislocation density induced by severe plastic deformation and the partial dissolution of Fe atoms into the Cu matrix [15,16], which severely hinders electron transport. Although aging treatment can promote the formation of Fe precipitates and increase the electrical conductivity to 24.8–47% IACS, the accompanying decrease in dislocation density and weakening of strengthening mechanisms [17] usually reduce the strength by 15–30% [18,19,20], making it difficult to reconcile the strength–conductivity trade-off. To overcome this bottleneck, researchers have attempted multiple cold rolling–aging cyclic processes. For example, Tang et al. [11] obtained a synergistic property combination of 554 MPa and 70% IACS through a four-step thermomechanical treatment, while Wang et al. [12] also achieved an excellent combination of 608 MPa and 58% IACS. However, these processes are lengthy, energy-intensive, and inefficient, which severely limits their large-scale industrial application.

Warm rolling has attracted increasing attention in recent years as a new route for thermo-mechanical regulation [21,22,23,24,25]. Xu et al. [25] warm-rolled Cu–10Fe at 400 °C and obtained a strength of 446 MPa and an electrical conductivity of 40% IACS, which were significantly superior to those achieved by conventional cold rolling (471 MPa/28% IACS). This advantage can be attributed to the enhanced diffusivity of Fe atoms, the formation of dispersed precipitates, and the introduction of partially recrystallized microstructures under warm-rolling conditions [26,27]. However, existing studies have mainly focused on the Cu–10Fe system. A systematic understanding is still lacking regarding Fe-phase evolution, Cu-matrix texture variation, KAM/GND response, and their coupling relationships with mechanical–electrical properties during warm rolling of alloys with higher Fe contents, such as 15% and 20% [28,29]. In particular, when the Fe content increases from 10% to 15% and 20%, the increased Fe-phase volume fraction and decreased interphase spacing may affect two-phase deformation compatibility, local stored-energy distribution, and dynamic recovery/local recrystallization behavior, which require further clarification. Most available studies have focused on Cu–10Fe or lower-Fe systems, while systematic investigations of Cu–Fe alloys with Fe contents of 15 wt.% and 20 wt.% remain limited. For high-Fe Cu–Fe alloys, increasing Fe content is expected to increase the volume fraction and interfacial area of the Fe-rich phase, which may strongly influence strain partitioning between the Cu-rich FCC matrix and Fe-rich BCC phase, rolling texture development, GND accumulation near Cu/Fe interfaces, and the final strength–ductility–conductivity balance. Therefore, this work aims to clarify the relationship among Fe-rich phase morphology, texture evolution, KAM/GND response, and mechanical/electrical properties during warm rolling of high-Fe Cu–Fe alloys.

In this study, three typical high-Fe-content Cu-Fe alloys, namely Cu-10Fe, Cu-15Fe and Cu-20Fe, were selected as the research objects. The inherent correlation mechanism among the Fe-rich phase morphology, KAM/GND response behavior, mechanical properties and electrical properties of high-Fe-content Cu-Fe alloys during the single-pass warm rolling process at 500 °C was systematically analyzed. This work is expected to provide theoretical support and process reference for the preparation of high-Fe-content Cu-Fe alloys, and promote the transformation of Cu-Fe alloys from laboratory basic research to engineering practical applications.

## 2. Materials and Methods

**Figure 1:**
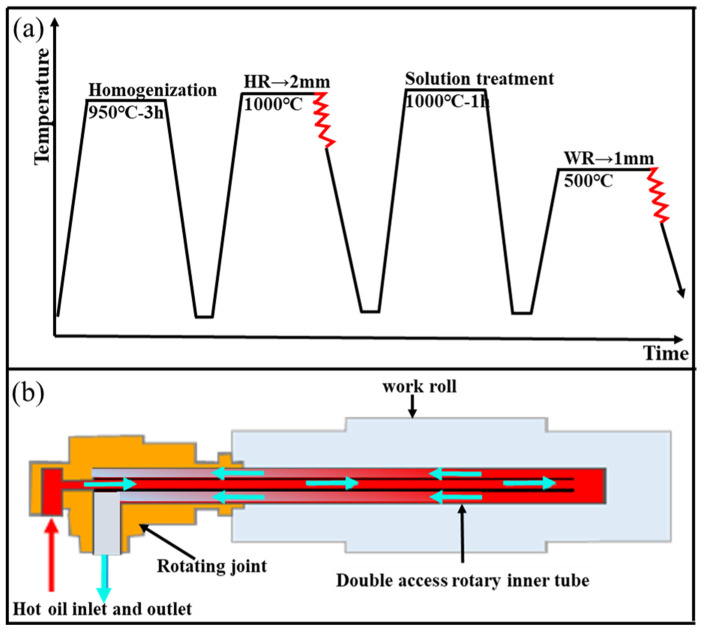
Process flow and equipment schematic diagram: (**a**) processing route; (**b**) schematic diagram of work roll heating.

Pure Cu and Cu-Fe master alloy were proportioned according to the designed compositions and melted in a vacuum melting furnace to prepare Cu-10Fe, Cu-15Fe, and Cu-20Fe alloy ingots (diameter 90 mm, height 110 mm), where Fe refers to the mass fraction. Chemical composition analysis showed that the Fe mass fractions in the three alloys were 10.3%, 15.4%, and 20.3%, respectively. The processing route is shown in Figure 1a. First, the Cu–Fe alloy ingots were homogenized at 950 °C × 3 h under a nitrogen atmosphere and then hot-rolled after heating at 1000 °C. After solution treatment at 1000 °C × 1 h, the hot-rolled plates were pickled and ground to remove the oxide layer, yielding plates with a thickness of 2 mm for warm rolling. Before warm rolling, the plates were heated in a furnace and then subjected to single-pass rolling at 500 °C with a 50% reduction. To reduce the temperature drop caused by contact between the plates and the work rolls, the rolls were preheated to 200 °C. A schematic diagram of the device is shown in Figure 1b. The thickness of the rolled samples was approximately 1 mm.

After mechanical polishing, microstructural observations were performed using an OLYMPUS-GSX500 optical microscope (OM, Olympus BX53M, Leica, Wetzlar, Germany) and a JEOL JXA8530F field-emission electron probe microanalyzer (EPMA, JXA-8530F, JEOL, Tokyo, Japan). The samples for EBSD analysis were mechanically polished and then argon-ion polished to obtain a flat, low-stress surface. The EBSD probe was a SYMMETRY S2 detector (EBSD, Symmetry S2, Oxford instruments, Abingdon, UK). All EBSD analyses in this study adopted CS0, the primary sample coordinate system. All electron backscatter diffraction (EBSD) measurements in this study were carried out on the longitudinal section parallel to the rolling direction (RD), i.e., the RD-ND plane. This section can fully characterize the grain orientation and substructure distribution features of the alloy after rolling deformation. The core test parameters are set as follows: the accelerating voltage is 20 kV, the probe current is stably controlled at 10 nA, the scanning step size is 0.1 μm, the scanning grid dimension is 600 × 500 pixels, and the total observation area is 60 μm × 50 μm. The orientation angular resolution of the whole system is better than 0.5°, which fully satisfies the accuracy requirements for subsequent KAM (Kernel Average Misorientation) and GND (Geometrically Necessary Dislocation) analyses. After raw data acquisition, only the standard 3 × 3 kernel noise filtering algorithm was applied to correct isolated misindexed points. No additional smoothing or interpolation was performed on the original orientation data, which maximally preserves the intragranular misorientation details and guarantees the accuracy of subsequent KAM and GND calculation results. EBSD data were used to obtain inverse pole figure (IPF) maps, recrystallized grain distribution maps, KAM maps, and GND density distribution. Recrystallized grains, substructured grains, and deformed grains were distinguished according to intragranular misorientation and grain-boundary characteristics. The dark-contrast regions observed in SEM are identified as Fe-rich domains, and the corresponding EBSD phase map was indexed using FCC Cu and BCC Fe as the target phases.

Electrical conductivity was measured using a ZY9987 digital micro-ohmmeter (Shanghai Zhengyang Instrument Factory, Shanghai, China), and the average of multiple measurements was taken as the final result. Mechanical properties were tested using a CMT5105 electronic universal testing machine (SHIMADZU, Kyoto, Japan) at a strain rate of 1 × 10^−2^ s^−1^. The specimen thickness was 1 mm, the gauge length was 15 mm, and the width was 3.5 mm. Three tensile specimens were tested for each condition, and electrical conductivity was measured at three different positions for each sample state.

## 3. Results

### 3.1. As-Cast Microstructure of Cu-Fe Alloys

**Figure 2:**
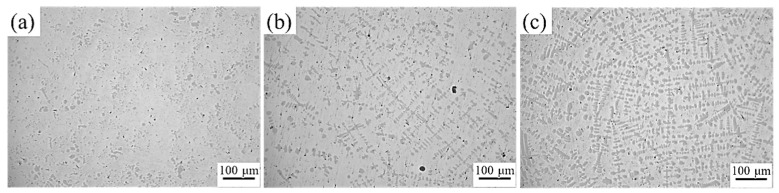
Metallographic image of as-cast microstructure of Cu-Fe alloy: (**a**) 10% Fe; (**b**) 15% Fe; (**c**) 20% Fe.

**Figure 3:**
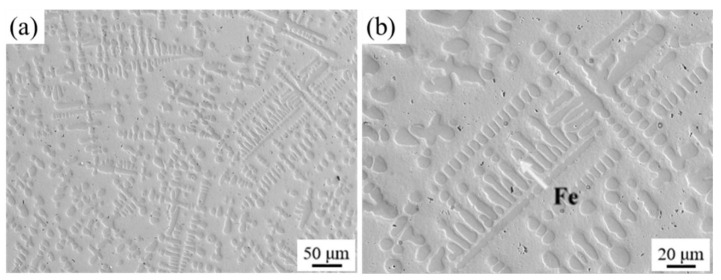
SEM image of as-cast microstructure of Cu-20Fe alloy: (**a**) 200×; (**b**) 500×.

Figure 2 shows the as-cast microstructures of the Cu–Fe alloys. For the Cu–10Fe alloy (Figure 2a), a large number of spherical or blocky Fe-rich phases were distributed in the Cu matrix, accompanied by a small amount of incompletely developed dendritic Fe-rich phase. The Fe-rich phase exhibited a wide size distribution. The diameter of small particles could be as low as approximately 4 μm, whereas the main trunk length of individual incompletely developed dendrites could reach several hundred micrometers. According to statistical analysis using Image Pro Plus software (Image-Pro Plus, Version 7.1, Media Cybernetics, Rockville, MD, USA), the average diameter of the Fe-rich phase was 13.86 μm. For the Cu–15Fe alloy (Figure 2b), the Fe-rich phase exhibited a relatively regular morphology and mainly existed in near-spherical cross-sectional features or rod-like forms. A small number of parallel rod-like Fe-rich phases had not yet fully developed into dendrites, and the average size was approximately 15.85 μm. For the Cu–20Fe alloy (Figure 2c), the Fe-rich phase was still dominated by near-spherical cross-sectional features or rod-like morphologies; however, the proportion of rod-like Fe-rich phase increased, and the dendritic features became more pronounced. Some parallel rod-like Fe-rich phases were closely connected with dendrite trunks, and the average size was approximately 20.09 μm. Figure 3 further presents the SEM morphology of the as-cast microstructure of the Cu–20Fe alloy shown in Figure 2c. It can be seen that the dendritic or fishbone-like Fe-rich phases observed in the metallographic images were actually composed of short rod-like Fe-rich phases. These short rod-like Fe-rich phases were arranged in parallel or perpendicular directions to form dendritic branches. Owing to the relatively high solidification rate, the short rod-like Fe-rich phases were retained before sufficient growth, thus forming incompletely developed dendritic structures.

In this study, the Cu–Fe alloys were mainly composed of near-spherical cross-sectional features Fe-rich phases, with dendritic Fe-rich phases as a secondary feature. This is because Cu–Fe alloys undergo two solidification processes [12]. In the first process, when the temperature decreases below the liquidus, the metastable Cu–Fe alloy undergoes liquid–liquid phase separation, forming Fe-rich and Cu-rich liquid phases. Due to liquid motion and convection, the Fe-rich liquid tends to form small dispersed droplets. Because the melting point of the Fe-rich liquid phase is higher than that of the Cu-rich liquid phase, the Fe-rich liquid solidifies first, initially in a spherical form, thereby forming spherical Fe-phase particles. In addition, owing to the Marangoni effect [30,31], larger Fe-rich droplets attract smaller Fe-rich microdroplets. If the small droplets are not completely absorbed by the larger droplets before solidification, small Fe-rich particles may form around some large spherical Fe-rich particles [32]. The second process occurs during the solidification of the Cu-rich liquid, in which Fe precipitates from the Cu melt to form Fe-phase nuclei. These nuclei grow spherically and rapidly become unstable, resulting in the formation of Fe-phase dendrites.

### 3.2. Solution-Treated Microstructure and Properties of Cu–Fe Alloys

**Figure 4:**
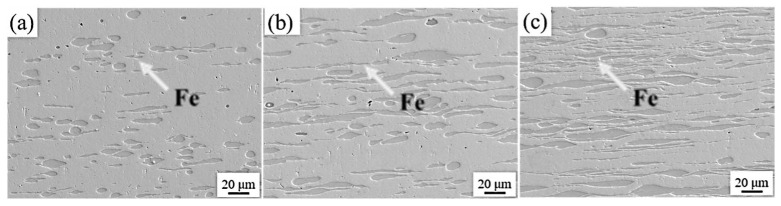
SEM images of solution-treated Cu-Fe alloys with different Fe contents: (**a**) 10% Fe; (**b**) 15% Fe; (**c**) 20% Fe.

Figure 4 shows the SEM microstructures of the Cu–Fe alloys after solution treatment, in which the darker regions correspond to the Fe-rich phase. As shown in Figure 4a, the Fe-rich phase in the Cu–10Fe alloy mainly exhibited rod-like or spherical morphologies, with only slight morphological changes compared with the as-cast Cu–10Fe alloy. Hot rolling and solution treatment mainly caused the Fe-rich phase to distribute along the rolling direction. A small number of rod-like Fe-rich phases were elongated into fibrous forms, whereas most spherical Fe-rich phases underwent only slight flattening deformation. As shown in Figure 4b, for the Cu–15Fe alloy, because the Fe-rich phase was already relatively concentrated in the as-cast microstructure, the interaction among Fe-rich phases during deformation affected the microstructure. Except for a few larger Fe-rich phases with relatively small deformation, most Fe-rich phases were elongated into fibrous structures along the rolling direction. As shown in Figure 4c, for the Cu–20Fe alloy, the Fe-rich phase was further fiberized and tended to be distributed in parallel. This was related to the parallel rod-like Fe-rich phases in the as-cast microstructure and their connection with dendrite trunks. During subsequent deformation, interactions among adjacent Fe-rich phases promoted the cooperative deformation of the Fe-rich phase along the rolling direction.

**Figure 5:**
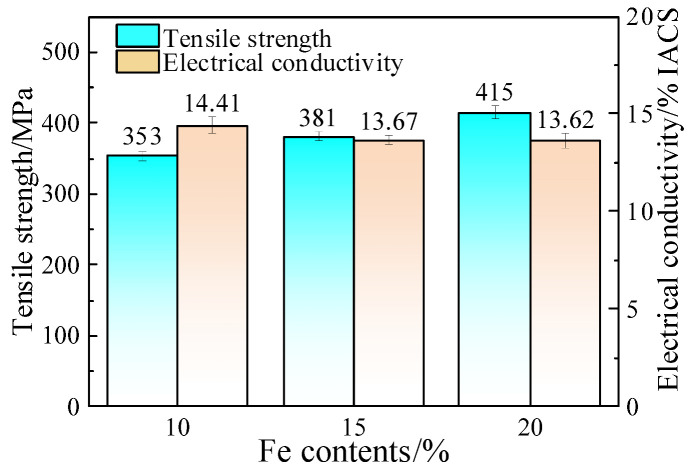
The properties of solution-treated Cu-Fe alloys with different Fe contents.

Figure 5 shows the ultimate tensile strength and electrical conductivity of Cu–Fe alloys with different Fe contents after solution treatment. The ultimate tensile strength and electrical conductivity of the Cu–10Fe alloy were 353 MPa and 14.41% IACS, respectively. When the Fe content increased to 15%, the ultimate tensile strength increased to 381 MPa, which was 7.34% higher than that of Cu–10Fe, while the electrical conductivity decreased to 13.67% IACS. When the Fe content was further increased to 20%, the ultimate tensile strength reached 415 MPa, corresponding to increases of 17.56% and 8.92% compared with Cu–10Fe and Cu–15Fe, respectively. The electrical conductivity was 13.62% IACS, which was close to that of Cu–15Fe.

### 3.3. Warm-Rolled Microstructure and Properties of Cu-Fe Alloys

#### 3.3.1. Warm-Rolled Microstructure of Cu-Fe Alloys

**Figure 6:**
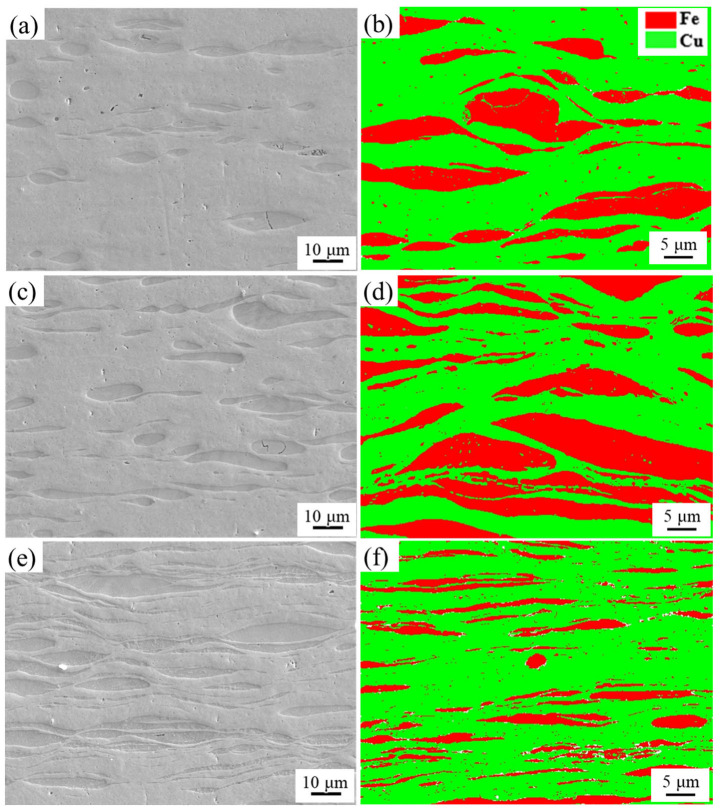
Microstructures and phase distribution maps of rolled Cu–Fe alloys: (**a**,**b**) Cu-10Fe, (**c**,**d**) Cu-15Fe, and (**e**,**f**) Cu-20Fe. Panels (**a**,**c**,**e**) show SEM images, and panels (**b**,**d**,**f**) show phase distribution maps, where red denotes the BCC Fe-rich phase and green denotes the FCC Cu matrix. The green (FCC) pixels observed within the red (BCC) regions originate from misindexed points caused by local surface relief, pattern overlap, low pattern quality, or mixed diffraction signals near the two-phase boundaries.

Figure 6 shows the SEM morphologies and phase distribution maps of Cu–Fe alloys with different Fe contents after single-pass warm rolling at 500 °C with a 50% reduction. In the SEM images, the darker regions correspond to the Fe-rich phase. In the phase distribution maps, the red regions represent the Fe-rich phase, and the green regions represent the Cu matrix. As shown in Figure 6a,b, the Fe-rich phases in the Cu–10Fe alloy were relatively far apart, and the spacing between some adjacent Fe-rich phases reached several tens of micrometers. The Fe-rich phase was mainly spherical or spindle-like, with limited elongation along the rolling direction. As shown in Figure 6c,d, the number of Fe-rich phases in the Cu–15Fe alloy increased, and the interphase spacing decreased. Most Fe-rich phase spacings were approximately more than ten micrometers, and some local regions were below 10 μm. Both spindle-like and fibrous Fe-rich phases were present, indicating a certain degree of inhomogeneity in Fe-rich phase deformation. As shown in Figure 6e,f, the Fe-rich phase in the Cu–20Fe alloy was more densely distributed, most Fe-rich phase spacings were below 10 μm, and the continuous fibrous features along the rolling direction became more pronounced. The average interphase spacing of the Fe-rich phase in three alloys was statistically analyzed via the intercept method. The obtained average interphase spacing of the second phase is 4.63 ± 0.5 μm, 3.65 ± 0.3 μm and 2.31 ± 0.3 μm, respectively. Similar to that in Figure 4 a large number of Fe-rich phases can also be observed in the microstructure after warm rolling.

**Figure 7:**
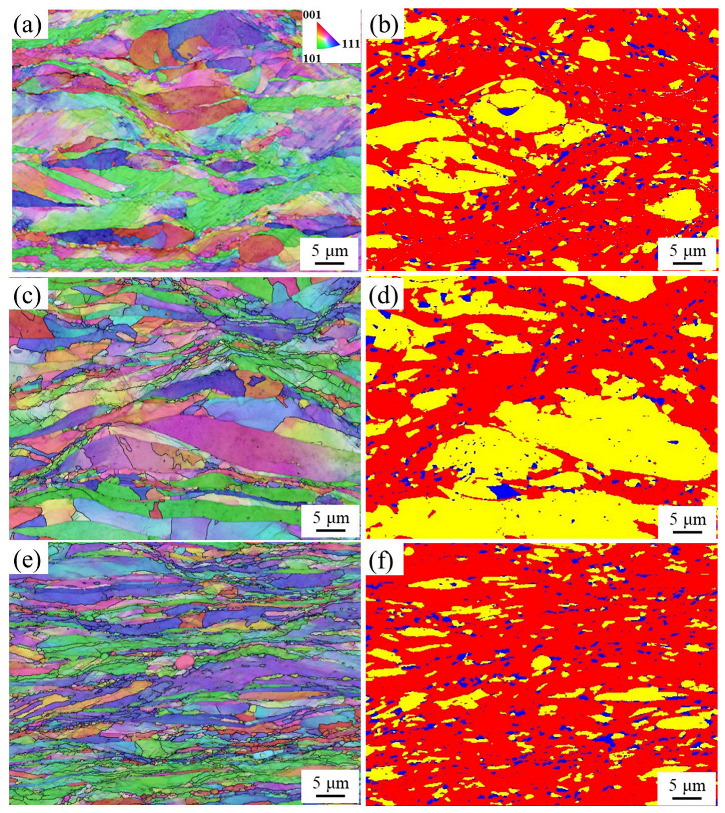
The EBSD analysis maps of rolled Cu-Fe alloys: (**a**,**c**,**e**) IPF map; (**b**,**d**,**f**) recrystallized grains. Red regions represent deformed grains, yellow regions indicate modified subgrains, and blue regions denote recrystallized grains.

Figure 7 shows the IPF maps and recrystallized grain distribution maps of Cu–Fe alloys with different Fe contents after single-pass warm rolling at 500 °C with a 50% reduction. In addition, the reference coordinate system for EBSD analysis is CS0. Recrystallized grains were identified via Grain Orientation Spread (GOS) analysis. Combined with the microstructural characteristics of Cu-Fe alloy, grains with GOS ≤ 2° were defined as fully recrystallized grains, those with GOS between 2° and 5° were classified as substructures, and grains with GOS ≥ 5° were identified as deformed grains with obvious residual strain. As shown in Figure 7a,c,e, the grains in the Cu–10Fe alloy were significantly elongated and underwent orientation changes after rolling, with local orientations dominated by <101>. With increasing Fe content, the grain orientation distribution became more dispersed, and the preferred orientation of the Cu matrix was weakened. Figure 7b,d,f show that deformed grains, substructured grains, and recrystallized grains coexisted in the alloys after warm rolling [33]. With increasing Fe content, the fraction of recrystallized grains generally increased, reaching 13.9% in the Cu–20Fe alloy. This indicates that dynamic recovery and local recrystallization occurred during warm rolling at 500 °C, although the degree of recovery/recrystallization differed with Fe content.

**Figure 8:**
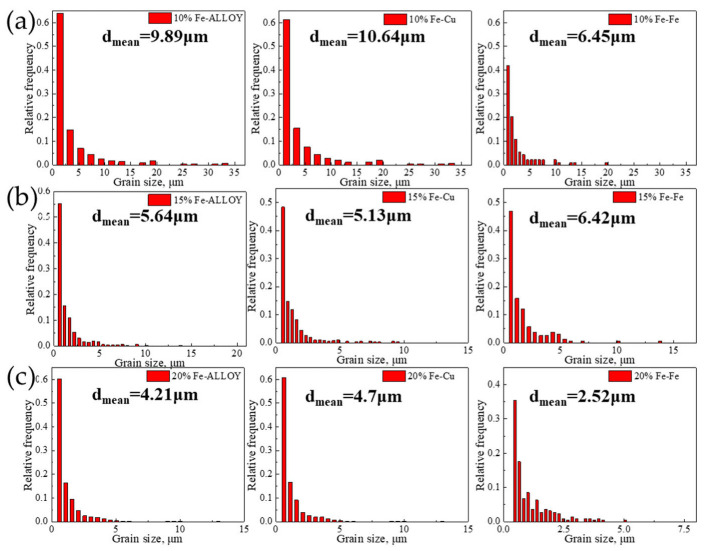
The grain size distribution of rolled Cu-Fe alloys: (**a**) 10% Fe; (**b**) 15% Fe; (**c**) 20% Fe.

Figure 8 shows the grain size distributions of Cu–Fe alloys with different Fe contents after warm rolling at 500 °C. For the Cu–10Fe alloy, because the Fe-rich phase was relatively discrete and the two-phase deformation compatibility was limited, the average grain size of the alloy was 9.89 μm. The average grain sizes of the Fe-rich phase and Cu matrix were 6.45 μm and 10.64 μm, respectively. When the Fe content increased to 15%, the Fe-rich phase distribution exhibited the coexistence of dense and sparse regions (Figure 6c,d). The average grain size of the Fe-rich phase remained almost unchanged at 6.42 μm, whereas the average grain size of the Cu matrix decreased to 5.13 μm, and the overall average grain size of the alloy decreased to 5.64 μm. When the Fe content was further increased to 20%, the spatial distribution of the Fe-rich phase became denser and the degree of fiberization [24] increased. The average grain size of the Fe-rich phase decreased significantly to 2.52 μm, while the overall average grain size of the alloy and the average grain size of the Cu matrix were 4.21 μm and 4.7 μm, respectively.

**Figure 9:**
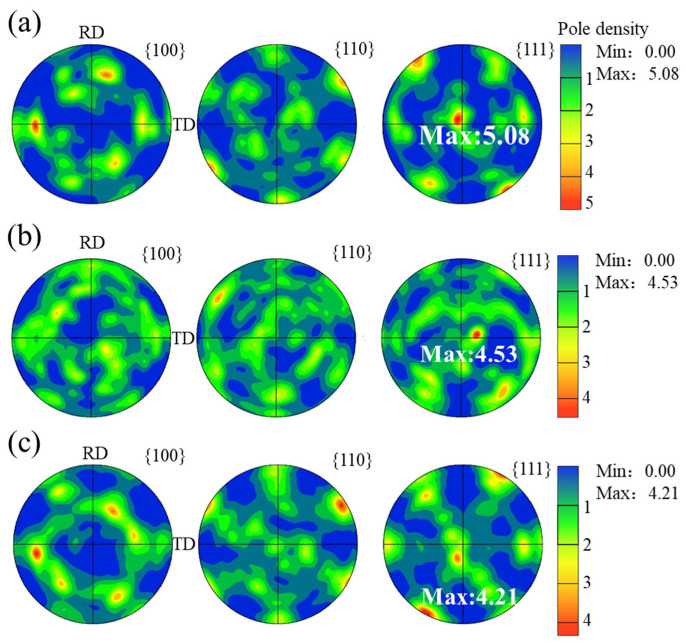
{100}, {110}, and {111} pole figures of Cu matrix in rolled Cu-Fe alloys: (**a**) 10% Fe; (**b**) 15% Fe; (**c**) 20% Fe.

Figure 9 shows the {100}, {110}, and {111} pole figures of the Cu matrix in Cu–Fe alloys with different Fe contents after warm rolling at 500 °C. As the Fe content increased from 10% to 20%, the maximum texture intensity of the Cu matrix decreased from 5.08 (Figure 9a) to 4.53 (Figure 9b) and 4.21 (Figure 9c), indicating that increasing Fe content weakened the preferred orientation of the Cu matrix during warm rolling. The rolling-deformation-related texture exhibited a weakening trend, suggesting that changes in Fe content and spatial distribution may have altered local strain partitioning and grain orientation selection in the Cu matrix [27,34,35].

Through analysis of pole figures, the average orientation density F of each sample was calculated as follows: 0.892 for Cu-10Fe, 0.903 for Cu-15Fe, and 0.918 for Cu-20Fe. The F value is a parameter obtained by uniform integration averaging of full-space orientation density measured from pole figures. It is generally accepted that the theoretical F value of a completely randomly oriented texture-free sample is 1; the closer F is to 1, the more uniform the overall grain orientation distribution and the weaker the preferred orientation of texture. The results show the following overall variation trend: as Fe content increases from 10% to 20%, the pinning hindrance effect of Fe second phase on Cu matrix grain rotation gradually increases, and the overall grain orientation distribution is more likely to be close to the random texture-free state. Accordingly, the F value, reflecting overall orientation uniformity, rises gradually and keeps approaching the theoretical texture-free value 1. Texture density reflects local orientation density, and the pole figure peak in this work also decreased gradually from 5.08 to 4.21. The variation rules of the F value and pole figure peak correspond to each other, proving that the increase in Fe content reduces the preferred orientation of Cu matrix.

#### 3.3.2. Property Changes in Warm-Rolled Cu–Fe Alloys

**Figure 10:**
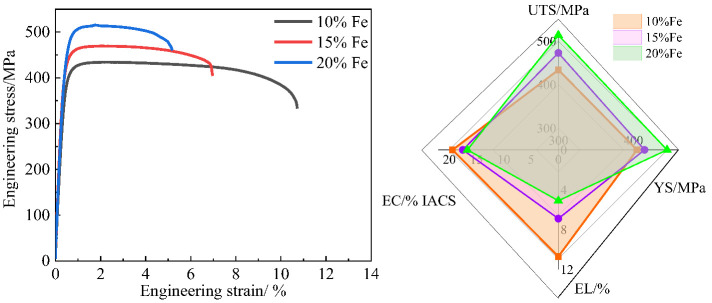
The variations in tensile properties and electrical conductivity of rolled Cu-Fe alloys.

Figure 10 shows the changes in tensile properties and electrical conductivity of Cu–Fe alloys with different Fe contents after warm rolling at 500 °C. When the Fe content was 10%, the ultimate tensile strength of the Cu–Fe alloy was 434 MPa, and the elongation was relatively high, reaching 10.7%. With increasing Fe content, the elongation decreased markedly. At an Fe content of 15%, the elongation was 6.9%, while the ultimate tensile strength of the Cu–Fe alloy increased to 471 MPa. When the Fe content increased to 20%, the elongation decreased to 5.1%, indicating a significant reduction in plasticity, although a certain degree of ductility was still retained. Whether brittle fracture occurred still needs to be further confirmed by fracture morphology. At this Fe content, the ultimate tensile strength of the Cu–Fe alloy increased to 514 MPa. As shown in Figure 10, the electrical conductivity of the Cu–Fe alloys decreased with increasing Fe content; however, it was much higher than that of the solution-treated samples. Compared with Figure 5, the electrical conductivity increased by 25.84%, 28.23%, and 22.68%, respectively, indicating that warm rolling reduced the adverse effect of increasing Fe content. As the Fe content increased from 10% to 20%, the electrical conductivity decreased from 19.43% IACS to 16.71% IACS.

**Figure 11:**
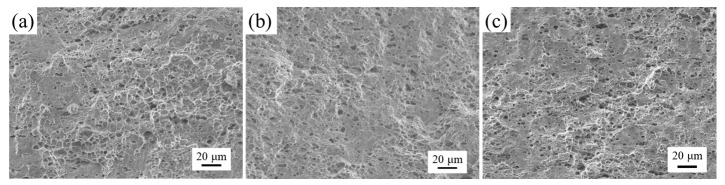
Fracture surface SEM images of rolled Cu-Fe alloys with different Fe contents: (**a**) 10% Fe; (**b**) 15% Fe; (**c**) 20% Fe.

As the Fe content increases from 10% to 15% and then to 20%, the overall toughness of the Cu-Fe alloy shows a gradual downward trend: as shown in Figure 11a, the dimples on the fracture surface of the Cu-10Fe alloy are larger in size and more uniformly distributed, with complete tear ridges, demonstrating the best plasticity and toughness performance; as shown in Figure 11b, the dimple size of the Cu-15Fe alloy is slightly reduced, but it still maintains a uniform distribution, and its toughness is slightly lower than that of the Cu-10Fe alloy; as shown in Figure 11c, the dimples of the Cu-20Fe alloy are generally finer, and the higher Fe content brings more nucleation sites for second-phase micropores, so the toughness is relatively lower.

## 4. Discussion

### 4.1. Effect of Fe Content on Warm-Rolled Microstructure of Cu-Fe Alloys

KAM can be used to evaluate the density of geometrically necessary dislocations (GND) during deformation. The KAM value is determined based on 24 neighboring points. During the calculation process, any local misorientation angles greater than 5° are excluded to eliminate the influence of interfering factors, such as high-angle grain boundaries, on the research results. The local misorientation at a point (100 nm × 100 nm) is determined using this point and its 24 surrounding points. Equation (1) can be employed to calculate the KAM value.

(1) $$△\theta_{i}=\frac{1}{n}\sum_{j=1}^{n}|\theta_{j}^{sur}-\theta_{i}|$$

where $\theta_{i}$ represents the local misorientation at point “*i*”. $\theta_{j}^{sur}$ represents the local misorientation at point “*j*”. *n* is the number of neighboring points involved in the calculation. Equation (2) can be employed to calculate the GND value.

(2) $$\rho GND=\frac{2\Delta \theta_{i}}{\mu b}$$

where $\theta_{i}$ represents the local misorientation at point “*i*”. *n* is the number of neighboring points involved in the calculation. *u* is the unit length of a point (100 nm); *b* is the Burgers vector.

**Figure 12:**
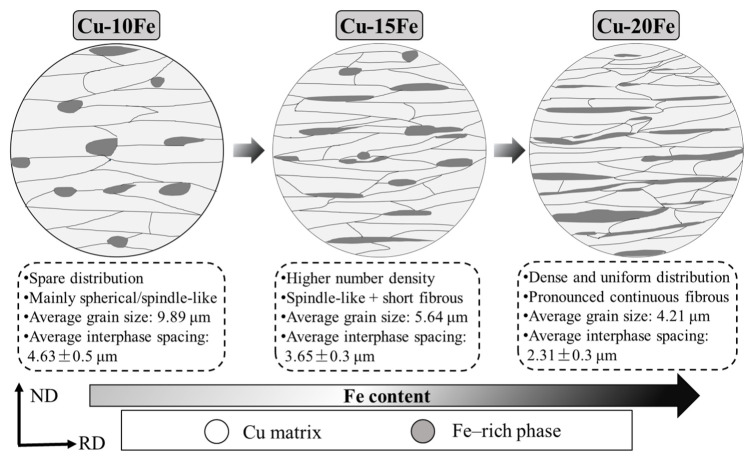
Schematic diagram of the morphological evolution of Fe-rich phase.

**Figure 13:**
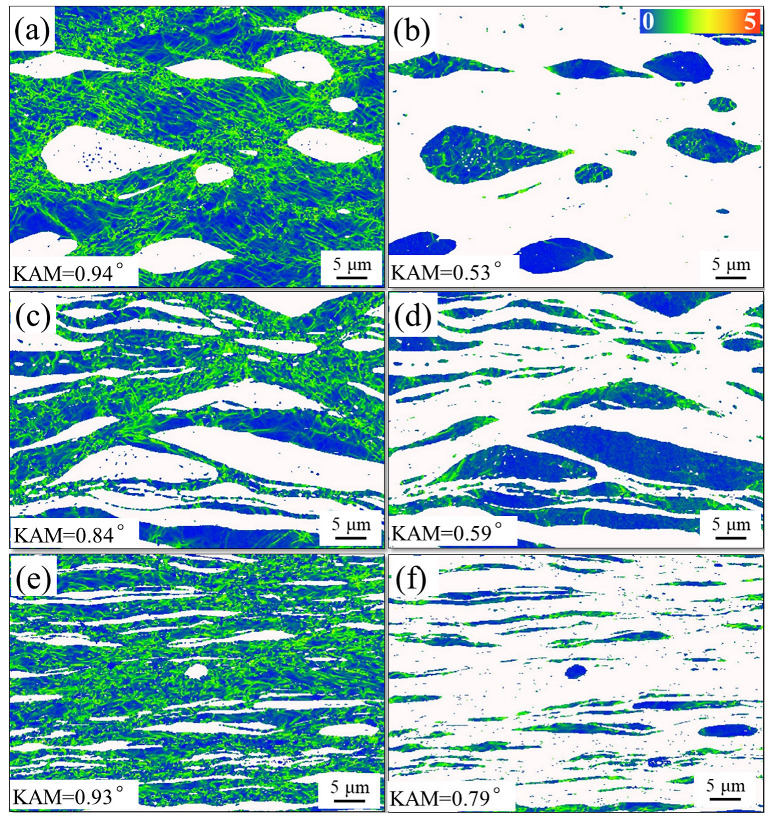
KAM maps of rolled Cu-Fe alloys: (**a**) 10% Fe; (**b**) 15% Fe; (**c**) 20% Fe. The color scale indicates local misorientation in degrees. (**a**,**c**,**e**) represent the Cu matrix, while (**b**,**d**,**f**) represent the Fe-rich phase.

**Figure 14:**
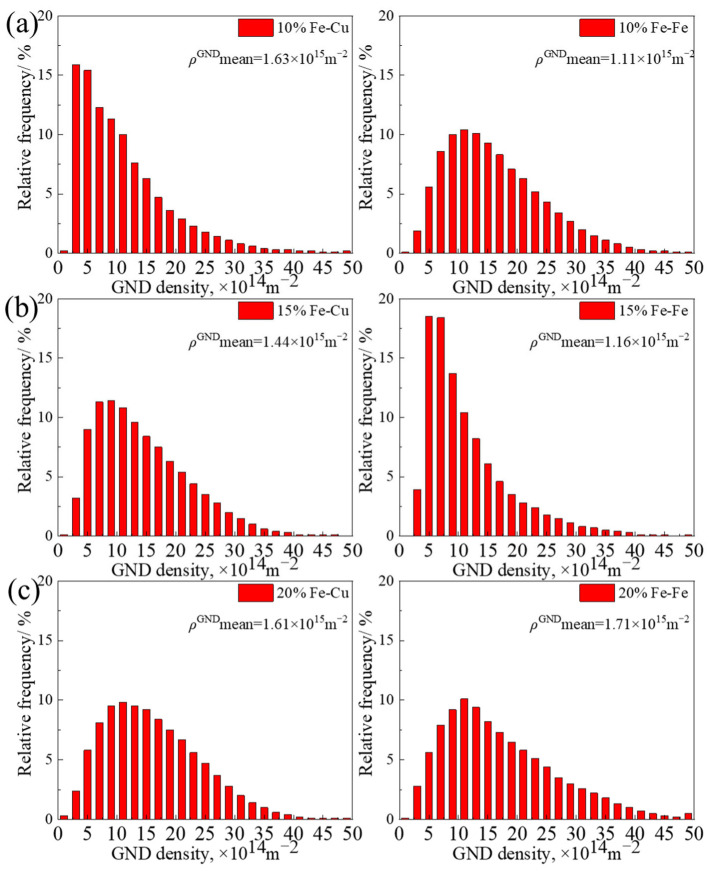
GND density distribution of rolled Cu-Fe alloys: (**a**) 10% Fe; (**b**) 15% Fe; (**c**) 20% Fe.

When the Fe content was 10%, the Fe-rich phase was mainly distributed in the Cu matrix as discrete spherical/spindle-like second phases. Because of the difference in mechanical properties between the Cu matrix and the Fe-rich phase, the deformation of the two phases was not fully coordinated during warm rolling (Figure 12). Plastic deformation was mainly concentrated in the Cu matrix and near the Cu/Fe interface. As shown in Figure 13a,b, the KAM maps indicate that the local misorientation of the Cu matrix was relatively high (0.94° and 0.84°), whereas that inside the Fe-rich phase was relatively low (0.53° and 0.59°), suggesting that the overall deformation degree of the Fe-rich phase was limited at this composition. Meanwhile, the GND density distribution (Figure 14a) shows that the GND density in the Cu matrix was higher than that in the Fe-rich phase, indicating that dislocation accumulation by EBSD-derived KAM and GND distributions near the Cu/Fe interface was one of the main sources of deformation stored energy. This deformation feature favored local dynamic recovery and recrystallization nucleation; therefore, the recovery/recrystallization regions in Figure 7b were mainly distributed near the Fe-rich phase.

When the Fe content increased to 15%, the number of Fe-rich phases increased, and dense and sparse Fe-rich phase regions coexisted in the microstructure (Figure 12). Under this condition, alloy deformation was affected not only by the Cu/Fe interface but also by interactions among adjacent Fe-rich phases. The KAM maps in Figure 13c,d show that misorientation concentration still mainly occurred near the phase boundaries, whereas the local misorientation inside the densely distributed fine Fe-rich phases increased (0.79°), indicating that the Fe-rich phase participated more extensively in plastic deformation. Because of the inhomogeneous distribution of the Fe-rich phase, local deformation also showed a certain degree of heterogeneity. This is an important reason why the average grain size of the Fe-rich phase changed only slightly in the Cu–15Fe alloy, whereas the average grain size of the Cu matrix decreased significantly. The thermally activated conditions provided by warm rolling promoted dislocation rearrangement and dynamic recovery, thereby reducing the texture intensity of the Cu matrix compared with that in the Cu–10Fe alloy.

When the Fe content was further increased to 20%, the Fe-rich phase became more uniformly distributed and the interphase spacing decreased significantly (Figure 12). The densely distributed Fe-rich phase enhanced the interaction among local stress fields, making the originally difficult-to-deform spherical or spindle-like Fe-rich phase more easily elongated along the rolling direction, thereby forming a relatively continuous fibrous structure (Figure 13e,f). Compared with the Cu–15Fe alloy, the average grain size of the Fe-rich phase in the Cu–20Fe alloy decreased to 2.52 μm, corresponding to a decrease of approximately 60.75%, indicating a significant enhancement of Fe-rich phase refinement and fiberization. The dense Fe-rich phase distribution also helped disperse local strain concentration in the Cu matrix, thereby weakening the preferred orientation of the Cu matrix and further reducing texture intensity.

### 4.2. Effect of Microstructure on Mechanical Properties and Electrical Conductivity

As shown in Figure 6 and Figure 7, the Fe-phase morphology and two-phase deformation compatibility differed significantly with Fe content, thereby affecting the mechanical properties of the warm-rolled alloys. For the Cu–10Fe alloy, the Fe-rich phase was relatively discrete, and the Cu matrix accommodated the main plastic deformation. Meanwhile, the coexistence of deformed grains and fine recrystallized grains in the Cu matrix [34] helped obtain relatively high elongation while maintaining a certain strength [35]. As the Fe content increased to 15% and 20%, the number of Fe-rich phases increased and the interphase spacing decreased. The densely distributed Fe-rich phase formed stronger barriers to dislocation motion and enhanced work hardening, resulting in an increase in the ultimate tensile strength from 434 MPa to 514 MPa. However, the increased Fe-rich phase content also reduced deformation compatibility and restricted plastic flow of the matrix, decreasing the elongation from 10.7% to 5.1%. Dynamic recovery and local recrystallization during warm rolling could partially alleviate the adverse effect of high Fe content on plasticity, allowing the Cu–20Fe alloy to retain limited ductility. The texture results also indicate that, with increasing Fe content, the texture intensity weakened and the preferred orientation of the Cu matrix decreased, which was beneficial for improving local deformation partitioning.

During the warm rolling process of Cu-Fe alloy, frictional internal stress plays a significant role in enhancing the alloy’s strength. The phase interfaces generated during warm rolling impede dislocation movement. Dislocations intersect each other during the glide process, leading to hardening. Although warm rolling does not cause as drastic an increase in grain boundaries as cold rolling does, it still alters the state of grain boundaries. These grain boundaries similarly hinder dislocation movement. Together, they effectively increase the resistance to dislocation glide by boosting frictional stress, thereby significantly enhancing the alloy’s strength and endowing the alloy with superior mechanical properties after warm rolling [36,37].

Electrical conductivity is mainly affected by electron scattering mechanisms. Compared with the solution-treated state, the electrical conductivity of the samples warm-rolled at 500 °C was significantly improved, which may be related to the reduction in partial dislocation density by dynamic recovery, the weakening of lattice distortion by local recrystallization, and the precipitation of some solute Fe atoms from the Cu matrix during warm rolling (the possible reduction in solute Fe content or possible Fe segregation/precipitation inferred from the improvement of electrical conductivity and the relevant literature [25]). As the Fe content increased from 10% to 20%, the Fe-phase volume fraction and interphase area increased, which theoretically enhanced electron scattering and reduced electrical conductivity. However, the effects of recovery/recrystallization and reduced solute Fe during warm rolling could partially offset this adverse effect. Therefore, the electrical conductivity of the warm-rolled Cu–Fe alloys decreased only from 19.43% IACS to 16.71% IACS, corresponding to a decrease of approximately 2.73% IACS, indicating that warm rolling helped mitigate the negative effect of increasing Fe content on electrical conductivity. However, the volume fraction of Fe phase still exerts a certain effect on electrical conductivity. After conversion, the volume fractions of Fe-rich phase in Cu-10Fe, Cu-15Fe and Cu-20Fe alloys are 11.47%, 16.82% and 22.03%, respectively. The calculation results demonstrate that, under the warm rolling process condition, there is an extremely strong negative linear correlation between the volume fraction of Fe-rich phase and the electrical conductivity of the alloy: for every 1% increase in the volume fraction of Fe phase, the electrical conductivity of the alloy decreases by approximately 0.292%IACS.

In this study, when the Fe content increased from 10% to 20%, the volume fraction of Fe phase and the area of phase interface did increase significantly. Theoretically, this would create stronger electron scattering sources and exacerbate the loss of electrical conductivity. However, the experimentally observed decrease in electrical conductivity (~2.73% IACS) is far lower than the prediction of the simple model. This deviation is consistent with the surface/interface-dominant mechanism revealed by the research [38]. During the warm rolling process at 500 °C, the dynamic recovery and local recrystallization not only reduce the dislocation density and relieve lattice distortion, but more importantly, optimize the microstructure of the Cu-Fe phase interface—making the interface more regular with lower defect density, which effectively reduces the electron scattering efficiency at the interface.

### 4.3. Effect of Fe Content on the Deformation Mechanism During Warm Rolling

During warm rolling of Cu–Fe alloys, as the Fe content increased from 10% to 15% and 20%, the microstructure and deformation behavior exhibited a transition from heterogeneous deformation dominated by the Cu matrix/interface to cooperative deformation involving the Fe-rich phase.

**Figure 15:**
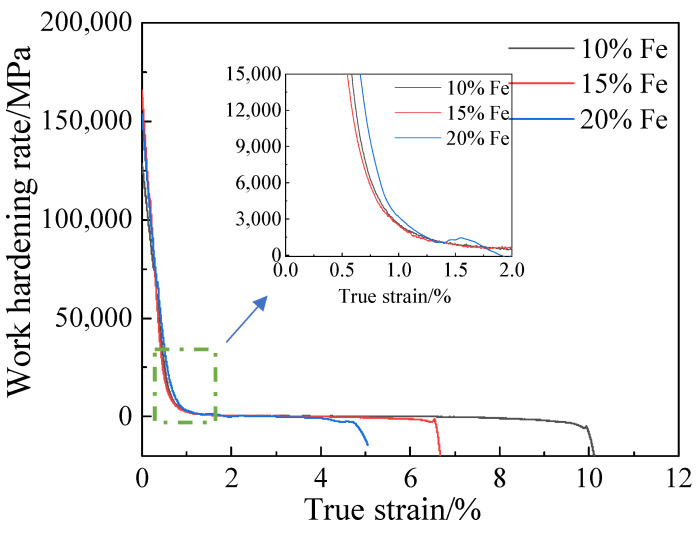
Work hardening curves of rolled Cu-Fe alloys.

In the Cu–10Fe alloy, the Fe-rich phase was distributed in the Cu matrix as discrete spherical/spindle-like second phases. Because the spacing between Fe-rich phases was relatively large (4.6 μm), interactions among adjacent Fe-rich phases were limited. Plastic deformation was mainly concentrated in the Cu matrix and near the Cu/Fe interface, while the deformation degree of the Fe-rich phase itself was relatively low. Under this condition, geometrically necessary dislocations (GNDs) were readily accumulated at the Cu/Fe interface, resulting in relatively high local deformation stored energy [39,40]. The GND distribution results show that the GND density in the Cu matrix was higher than that in the Fe-rich phase (Figure 14a), indicating that the alloy deformation at this composition was mainly characterized by interface-GND-dominated heterogeneous deformation. Combined with Figure 15, from the perspective of work hardening behavior, this interface-dominated heterogeneous deformation will lead to a higher initial work hardening rate: dislocations rapidly accumulate at Cu/Fe interfaces in the early deformation stage, the dislocation multiplication rate is much higher than the annihilation rate, and the work hardening effect is rapidly amplified.

When the Fe content increased to 15–20%, the Fe-phase volume fraction increased and the interphase spacing decreased (3.6 μm and 2.3 μm). The local stress fields among adjacent Fe-rich phases overlapped, promoting more pronounced plastic elongation and fiberization of the Fe-rich phase along the rolling direction [41]. At this stage, alloy deformation was no longer dominated solely by the Cu matrix and Cu/Fe interface, and the Fe-rich phase participated more significantly in overall plastic deformation. The GND distribution shows that, with increasing Fe content, the GND density in the Fe-rich phase gradually increased. In the Cu–20Fe alloy, both the Fe-rich phase and Cu matrix maintained relatively high dislocation densities (Figure 14b,c), indicating that the two phases jointly participated in deformation stored-energy accumulation [42,43,44]. Corresponding to the characteristics of the work hardening curve, this two-phase synergistic deformation will make the initial work hardening rate gradually decrease with the increase in Fe content: dislocation multiplication is distributed between the Cu matrix and Fe-rich phase, which makes the dislocation distribution more dispersed and weakens the initial hardening effect. Meanwhile, the higher the Fe content, the larger the volume fraction of the Fe-rich phase, and more interfaces become dislocation pile-up cores. Unlike severe cold rolling, which relies on large accumulated strain to promote Fe-rich phase fiberization, warm rolling at 500 °C in this study reduced the accumulated strain required for cooperative deformation of the Fe-rich phase through thermally activated dislocation climb, multiple slip, and dynamic recovery. Therefore, the decrease in interphase spacing caused by increasing Fe content and the thermally activated effect of warm rolling jointly promoted the transition from interface-dominated heterogeneous deformation to cooperative deformation involving the Fe-rich phase.

## 5. Conclusions

(1) After single-pass warm rolling at 500 °C with a 50% reduction, as the Fe content increased from 10% to 20%, the Fe-rich phase in the Cu–Fe alloys became more densely distributed, the degree of fiberization along the rolling direction was significantly enhanced, and the average grain size of the alloy decreased. Changes in the spatial distribution and morphology of the Fe-rich phase improved the two-phase deformation compatibility and weakened the preferred orientation of the Cu matrix, resulting in a decreasing trend in the texture.
(2) Increasing Fe content increased the ultimate tensile strength of the Cu–Fe alloys from 434 MPa to 514 MPa, while the elongation decreased from 10.7% to 5.1%. The densely distributed Fe-rich phase contributed to strength improvement but also intensified plasticity loss. Meanwhile, dynamic recovery and local recrystallization during warm rolling partially alleviated the adverse effect of high Fe content on plasticity, allowing the Cu–20Fe alloy to retain limited ductility. The electrical conductivity decreased from 19.43% IACS to 16.71% IACS, indicating that warm rolling could reduce the negative effect of increasing Fe content on electrical conductivity.
(3) KAM/GND analysis indicates that Fe content changed the two-phase deformation partitioning during warm rolling. In the Cu–10Fe alloy, plastic deformation was mainly concentrated in the Cu matrix and near the Cu/Fe interface, showing heterogeneous deformation dominated by interfacial GNDs. When the Fe content increased to 15–20%, the Fe-phase spacing decreased and more pronounced fiberization occurred. The deformation behavior of the alloy exhibited a transition toward cooperative deformation involving the Fe-rich phase.

## Data Availability

The original contributions presented in this study are included in the article. Further inquiries can be directed to the corresponding author.
